# Near-Real-Time Surveillance of Illnesses Related to Shellfish Consumption in British Columbia: Analysis of Poison Center Data

**DOI:** 10.2196/publichealth.8944

**Published:** 2018-02-23

**Authors:** Victoria Wan, Lorraine McIntyre, Debra Kent, Dennis Leong, Sarah B Henderson

**Affiliations:** ^1^ Environmental Health Services British Columbia Centre for Disease Control Vancouver, BC Canada; ^2^ British Columbia Drug and Poison Information Centre Vancouver, BC Canada

**Keywords:** poison control centers, public health surveillance, shellfish poisoning, norovirus, Vibrio parahaemolyticus

## Abstract

**Background:**

Data from poison centers have the potential to be valuable for public health surveillance of long-term trends, short-term aberrations from those trends, and poisonings occurring in near-real-time. This information can enable long-term prevention via programs and policies and short-term control via immediate public health response. Over the past decade, there has been an increasing use of poison control data for surveillance in the United States, Europe, and New Zealand, but this resource still remains widely underused.

**Objective:**

The British Columbia (BC) Drug and Poison Information Centre (DPIC) is one of five such services in Canada, and it is the only one nested within a public health agency. This study aimed to demonstrate how DPIC data are used for routine public health surveillance in near-real-time using the case study of its alerting system for illness related to consumption of shellfish (ASIRCS).

**Methods:**

Every hour, a connection is opened between the WBM software Visual Dotlab Enterprise, which holds the DPIC database, and the R statistical computing environment. This platform is used to extract, clean, and merge all necessary raw data tables into a single data file. ASIRCS automatically and retrospectively scans a 24-hour window within the data file for new cases related to illnesses from shellfish consumption. Detected cases are queried using a list of attributes: the caller location, exposure type, reasons for the exposure, and a list of keywords searched in the clinical notes. The alert generates a report that is tailored to the needs of food safety specialists, who then assess and respond to detected cases.

**Results:**

The ASIRCS system alerted on 79 cases between January 2015 and December 2016, and retrospective analysis found 11 cases that were missed. All cases were reviewed by food safety specialists, and 58% (46/79) were referred to designated regional health authority contacts for follow-up. Of the 42% (33/79) cases that were not referred to health authorities, some were missing follow-up information, some were triggered by allergies to shellfish, and some were triggered by shellfish-related keywords appearing in the case notes for nonshellfish-related cases. Improvements were made between 2015 and 2016 to reduce the number of cases with missing follow-up information.

**Conclusions:**

The surveillance capacity is evident within poison control data as shown from the novel use of DPIC data for identifying illnesses related to shellfish consumption in BC. The further development of surveillance programs could improve and enhance response to public health emergencies related to acute illnesses, chronic diseases, and environmental exposures.

## Introduction

### Background

Poison centers across the United States, Canada, Europe, Australia, and New Zealand have been long recognized for the direct patient care and educational programs that fulfill their core mandate [1-5]. The services provided by specialized pharmacists, nurses, and physicians at these centers typically include telephone consultations available 24 hours per day, every day of the year. Calls come from homes, workplaces, hospitals, and other health care facilities in urban, rural, and remote settings. Beyond their primary mandate to support the general public and health care professionals, poison centers also provide essential expertise in the field of toxicology through published articles and community outreach. Several studies in the United States have explored the direct impact of poison centers on avoided emergency room visits, physician visits, ambulance services, and other medical treatments [6-11]. A recent review found that every US $1 spent on poison centers translated to US $8 saved on unnecessary health care costs [12]. These studies also demonstrated that consultation with poison centers was associated with decreased hospital admissions for poisonings treated in emergency departments [7-11,13].

Public health concern over poison exposures is increasing because there are more potentially toxic products entering the consumer market [14-17], more pharmaceuticals being prescribed [18,19], new illicit drugs becoming available [19,20], and more toxins in the ambient environment [14,16,17]. Intentional and unintentional poison exposures are caused by hazardous mixtures of chemical, physical, and biological agents that affect humans and animals. Poisonings are the third leading cause of mortality from unintentional injury in Canada, behind motor vehicle crashes and falls [21]. One key tool in poison prevention is routine surveillance to identify long-term trends, short-term aberrations from those trends, and near-real-time changes in the occurrence of high risk cases. However, such surveillance is typically within the mandate of public health agencies and not within the mandate of poison centers. As such, strong partnerships are needed to fully harness the surveillance potential of data from poison centers.

Development of these partnerships has been prioritized in the United States, Europe, and New Zealand, where poison data have increased the capacity of public health surveillance to address morbidity and mortality from poisonings. Specifically, these systems have informed the response to food-borne illness [22,23], substance abuse [24], pharmaceutical misuse [25], and aquatic toxins [26]. They have also improved monitoring of covert threats [3] and mass public gatherings [27]. In the United States, much of this work is accomplished through the National Poison Data System (NPDS) [28], which provides nation-wide surveillance of exposures in near-real-time using a centralized database maintained by the American Association of Poison Control Centers. There is no equivalent infrastructure in Canada, and there is no routine review of the national data at predefined intervals [2]. Indeed, several recent reports comment on the reactive rather than proactive use of Canadian poison data to address specific issues, such as the meltdown of the nuclear reactors in Fukushima, Japan [29], deaths from contaminated ecstasy [30], and food-borne illnesses [31,32]. All of these examples highlight the potential for more systematic use of Canadian poison data for surveillance at the provincial and national scales.

### Canadian Poison Centers

There are 5 poison centers in Canada, all but one of which services more than one province or territory. In addition, 4 of these centers are associated with acute care facilities, but the British Columbia (BC) Drug and Poison Information Centre (DPIC) is located at the BC Centre for Disease Control (BCCDC) [2]. As a provincial public health agency, the BCCDC has the mandate and the resources necessary to conduct routine surveillance for a wide range of acute illnesses, chronic diseases, and environmental exposures. This mandate and these resources were extended to DPIC when it joined the BCCDC in 2011, making it possible to use the poison data for regular and systematic reporting to internal and external stakeholders. Since then, DPIC and the BCCDC have developed multiple surveillance systems related to different toxic exposures that operate on various time scales.

In late 2014, the research team launched the near-real-time alerting system for illness related to the consumption of shellfish (ASIRCS), which detects new cases within 1 hour of DPIC receiving the call. This was our first near-real-time system, and we highlight it here because (1) all cases of paralytic shellfish poisoning (PSP) are reportable under the Public Health Act of BC, (2) the automated system replaced a manual system with the same objectives, (3) shellfish-related illnesses are not limited to a particular demographic group or a particular time of year, and (4) it can easily be adapted to other applications or data from other poison centers.

Shellfish contaminated with bacteria, viruses, toxins, or some combination of the three can cause severe illness in those who consume them, and the incidence of such cases has been rising in BC over the past decade [33-35]. Examples of harmful contaminants include norovirus, *Vibrio parahaemolyticus* (*VP*), and different toxins that can accumulate in bivalve shellfish during harmful algal blooms.

Outbreaks related to all types have been reported in BC. In 2010, a norovirus outbreak caused 36 laboratory-confirmed illnesses among raw oyster consumers [33]. In 2015, an outbreak of *VP* caused acute gastroenteritis in 73 documented cases, which led to a ban on the sale of raw oysters in grocery stores and restaurants across the greater Vancouver area [34]. Estimates suggest there are approximately 350 unreported cases of infectious enteric illness for every reported case in BC [36]. In comparison with infectious agents, cases of toxic shellfish poisoning are rarer [37]. In 2011, an outbreak of diarrhetic shellfish poisoning (DSP) caused 60 illnesses in people who ate cooked mussels [35]. There have been 4 documented cases of PSP [38], which can cause paralysis, respiratory failure, and death [39]. Finally, concentrations of domoic acid, the toxin responsible for amnesic shellfish poisoning (ASP), have been increasing along the BC coast in recent years [40]. Although BC has not reported an outbreak related to ASP, the Canadian Food Inspection Agency notified the BCCDC of one case in 2016, and DPIC received a call from another suspected exposure involving three individuals in the same year.

The concept of reportable or notifiable diseases is fundamental to the practice of public health. Such diseases are considered to be of sufficient risk to the entire population that any cases diagnosed by doctors or laboratories much be reported to some central authority, often by law. These centralized data can then be used to identify and track outbreaks, and to inform public health decisions. Bacterial enteric illnesses are typically reported by a laboratory to a health authority following clinically relevant results, such as a positive test in stool or blood.

However, there are no clinical tests for shellfish biotoxins, and illnesses can only be confirmed if the toxin is detected in leftover products. This limitation means that most illnesses are reported as probable based on the symptoms collected by doctors. As there are no laboratory results, there is no systematic mechanism by which to report these illnesses to the responsible health authorities.

Sometimes there may be direct communication by doctors or individuals who are ill, but this is rare. Thus, it is challenging for health authorities to meet their duty to report cases of PSP or other shellfish toxins. Given that (1) DPIC receives calls about shellfish-related illnesses and is located within the BCCDC, and (2) the BCCDC has a responsibility to support mandatory reporting of PSP and other shellfish-related illnesses, the BCCDC decided to develop a system that would identify, isolate, and alert on all relevant cases. Here, we describe how ASIRCS works, the cases it identified in 2015 and 2016, and the public health actions related to those cases.

## Methods

### Study Area

The province of BC is located on the west coast of Canada, and it has more than 25,000 km of coastline between its mainland and island areas [41]. These coastal waters have always been an important source of fish and shellfish for the First Nations of Canada [42]. In more recent history, they have played an important role in broader subsistence, recreational, and commercial fisheries. For example, BC oysters account for approximately 60% of all Canadian oyster production, which had a market value of CAN $27.3 million in 2013 [43]. The wholesale value of the entire BC shellfish industry was estimated at CAN $279 million in 2015 [44]. The 2015 population of BC was approximately 4.7 million residents, the majority of whom live in close proximity to the coast in greater Vancouver (2.3 million), on Vancouver Island (767,000), or elsewhere (186,000) [45].

### How the Drug and Poison Information Centre Operates

The DPIC service has a dedicated 24×7 phone line that provides poison information to the general public and health care professionals, including doctors, nurses, and emergency medical personnel in the province of BC and the Yukon Territory. Poison information services are provided by pharmacists and nurses certified as poison information specialists. When someone calls the poison line, the responding DPIC specialist gathers the information necessary to assess the urgency of the concern, and then begins to obtain a complete history by following set guidelines. In some cases, the exposed individual makes the call, but in the other cases, the caller has not been exposed. Furthermore, a single caller may be calling about an incident in which multiple people were exposed.

In some situations, the single call will automatically generate a separate record for each exposed individual, meaning that the number of calls DPIC receives is smaller than the number of cases DPIC manages. Once there is a full understanding of the exposure, the DPIC poison specialists triage the exposed individual or individuals to a health care facility if needed or provide home or on-site management recommendations where appropriate.

Depending on the nature of the exposure, the poison specialists routinely use their clinical experience and judgment along with other sources of toxicological information such as the Poison Management Manual [46] and Micromedex Solutions [47] to manage cases. Severe or unusual exposures may require follow-up calls to the treating health care facility until symptoms have subsided or life-threatening conditions have been resolved. All follow-up information is included in the single record for each case.

Since October 2011, DPIC has maintained records of all cases using the WBM software Visual Dotlab Enterprise (VDLE) [48], an electronic system specifically designed for use by poison centers. Like many other poison centers, DPIC staff use SAP Crystal Report XI Developer [49] to generate in-house summaries of data in VDLE, but this system lacks the analytic capacity to support routine surveillance. All of the DPIC surveillance systems, including ASIRCS, have been built in the R statistical computing environment [50], using the *RODBC* package [51] to interface with the VDLE database. Working with the data in R allows easy and flexible processing, manipulation, analysis, tabulation, plotting, and reporting on data collected by DPIC.

### How the Alerting System for Illness Related to the Consumption of Shellfish Works

Every hour on the hour, ASIRCS connects to the VDLE database and extracts all cases from the past 24 hours. The system uses a 24-hour period rather than a 1-hour period because it sometimes takes several hours for a case to be closed within VDLE. Case information is then extracted and merged from the relevant raw data tables before data cleaning.

The multistep procedure of data cleaning is challenging because it requires the transcription of information captured by the DPIC poison specialist into a standardized tabular format. For example, it is particularly challenging to extract relevant information from free-text fields in VDLE, where poison specialists document history, assessment, and recommendations as in a medical chart. Once all the data have been extracted and merged, ASIRCS scans the data for cases related to the consumption of shellfish based on criteria established by BCCDC food safety specialists.

An alert is generated when the following criteria are met:

- The case originated from within BC. At the time of the consultation, the DPIC poison specialists enter a city or postal code for the caller location, and this is georeferenced in the data cleaning stage.
- The case was about a human subject. This information is collected upon initial contact between the caller and the DPIC poison specialists and is entered into VDLE via a drop-down menu.
- The case was about a specific exposure rather than as a general inquiry. This information is entered into VDLE via a dropdown menu.
- The case was classified as “unintentional/food poisoning,” “adverse reaction/food,” or “unintentional/general.” This information is recorded by the DPIC poison specialists and is entered into VDLE via a dropdown menu following the NPDS Coding User Manual [52].
- Clinical free-text notes about the case contained one or more of the following words in a singular or plural form: clam, mussel, oyster, shellfish, paralytic, or neurolytic.
- The case has not been previously alerted. This is evaluated within ASIRCS by comparing the cases within the past 24 hours with the complete list of calls that have already alerted using their VDLE unique case identifiers.

### Public Health Follow-Up

When new cases are detected, ASIRCS generates an automated report tailored to the needs of the BCCDC food safety specialists (Multimedia Appendix 1). The file is saved in a secure location to protect personal privacy, and ASIRCS sends an automatic email to system users to let them know that an alert has been generated. The BCCDC food safety specialists review the alerted case or cases and use the information along with their expertise to assess whether the case or cases should be referred to the most responsible of the 5 regional health authorities for potential reporting. Once referred, the environmental health officers contact the patient or patients to collect further information about the case or cases, provided that consent and contact information for such follow-up was given at the time of the call to DPIC.

### Summary of Alerted and Missed Calls

Beta testing of ASIRCS started in late 2014, and we now have complete data for both 2015 and 2016. Here, we have reviewed all cases identified by the system over these 2 years and summarized them with respect to the nature of the exposure and public health follow-up. Specifically, we have identified those that were referred to the regional health authorities, and we have identified potential exposures to each group of shellfish toxins: PSP, ASP, or DSP. The cases were categorized into these 3 groups based on the reported symptoms matching each of the shellfish toxin exposure: numbness and tingling are indicative of PSP; headache, confusion, and disorientation are indicative of ASP; and diarrhea and abdominal cramps are indicative of DSP when symptom onset is <10 hours. Norovirus, *VP*, and other enteric illnesses may be considered when symptom onset is >10 hours.

Finally, we retrospectively checked all VDLE records from the same 2 years to evaluate whether we missed any cases that should have generated alerts. First, we applied the same criteria described above to all cases in the VDLE database and compared the results with the list of alerted cases. Second, we added the keyword “seafood” to the criteria above to evaluate whether extension of the keyword list would provide improved results.

## Results

**Figure 1:**
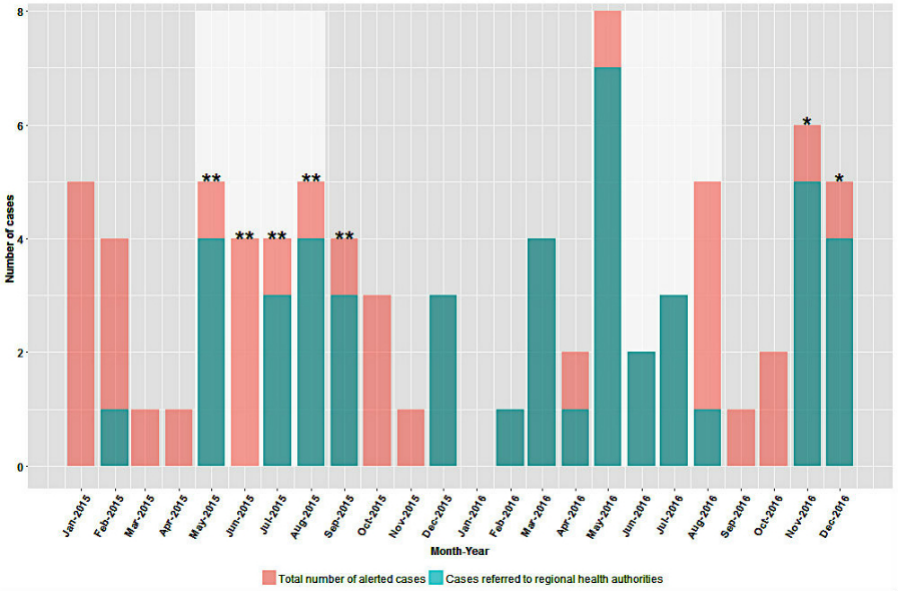
Time series of the total number of cases identified by the alerting system for illnesses related to consumption of shellfish, and the number of cases referred to regional health authorities from January 2015 to December 2016. The white areas indicate summer months, ** indicates a Vibrio parahaemolyticus outbreak, and * indicates a norovirus outbreak.

The ASIRCS system identified 79 cases from January 2015 to December 2016, 50% (40/79) of the cases were in 2015 and 49% (39/79) were in 2016. Approximately one-half of the cases occurred during the summer months of May to August in both years (Figure 1).

Consent for follow-up and the appropriate contact information were received by the DPIC poison specialists in all but 8% (6/79) of the cases. Of the 79 ASIRCS-alerted cases, 58% (46/79) were referred to the most responsible regional health authority for follow-up, but there was a difference between years. Of the 40 cases in 2015, 45% (18/40) were referred and 13% (5/40) were missing the necessary follow-up information. Of the 39 cases in 2016, 72% (28/39) were referred and none were missing follow-up information (Figure 1).

**Figure 2:**
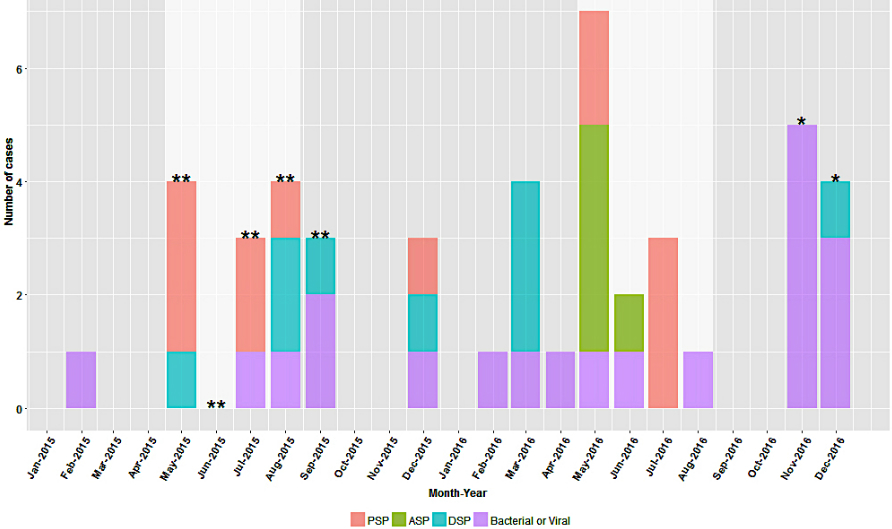
Time series of number of cases classified as paralytic shellfish poisoning (PSP), amnesic shellfish poisoning (ASP), diarrhetic shellfish poisoning (DSP), or bacterial or viral from January 2015 to December 2016. The white areas indicate summer months, ** indicates a Vibrio parahaemolyticus outbreak, and * indicates a norovirus outbreak.

Of the 46 cases referred to local health authorities, 57% (26/46) were classified as potential toxic shellfish poisonings because of PSP, ASP, or DSP (Figure 2), and Norovirus or *VP* was indicated in the remaining 44% (20/46) of the cases. Several of these cases coincided with a *VP* outbreak that occurred in the summer of 2015 [54] and a norovirus outbreak that began in November 2016 [53]. Of the 79 cases identified by ASIRCS, 42% (33/79) were not referred to regional health authorities following review by BCCDC food safety specialists.

Of these, 15% (5/33) would have been referred, but consent for follow-up was not provided. Among the remaining 85% (28/33) cases, some were because of allergic reactions and some were because of identification of nonshellfish-related cases from the keywords in the search algorithm. For example, at least one call related to oyster *mushrooms* (rather than bivalve mollusks) was flagged.

Retrospective validation found that ASIRCS missed 2 cases in 2015 and 9 cases in 2016, mostly because of system errors after updates to the R code. Delayed review of these cases by BCCDC food safety specialists found 1 (50%) of the 2 cases would have been referred to regional health authorities in 2015, and 5 (56%) of the 9 cases would have been referred in 2016. Of those cases that would have been referred, 33% (2/6) would have been classified as PSP, 17% (1/6) would have been classified as DSP, and 50% (3/6) would have been classified as bacterial or viral foodborne illnesses. When we included the additional keyword for “seafood,” an additional 11 cases were identified. Upon review by BCCDC food safety specialists, 55% (6/11) were related to foodborne illness associated with seafood, and 9% (1/11) may have been classified as a suspect bivalve biotoxin shellfish poisoning based on the details recorded during the call.

## Discussion

During its first 2 years of operation, ASIRCS identified 79 cases, with an equal number in each year. Of these cases, 58% (46/79) were referred to the most responsible regional health authority for follow-up. A total of 11 cases that should have been referred were not referred either because of missing contact information (5 cases, 45%) or failure of ASIRCS to alert (6 cases, 55%). More calls were referred in 2016 than in 2015, possibly reflecting greater presence of shellfish-related illness and ascertainment of more complete contact information collected by DPIC poison specialists at the time of call. On the basis of symptoms recorded in clinical notes, 20% (9/46) referred cases were classified as DSP, 26% (12/46) were classified as PSP, 11% (5/46) were classified as ASP, and 44% (20/46) were classified as bacterial or viral.

The automated, systematic elements of ASIRCS allow BCCDC food safety specialists to refer cases of shellfish-related illnesses to the appropriate reporting authorities within hours of DPIC receiving the call. The response time allows for rapid public health action, particularly during outbreaks of infectious or toxic agents. We chose to highlight ASIRCS in this report because it replaced an older, multistep system that was prone to human error and oversight (Figure 2). The old method required DPIC poison specialists to personally inform BCCDC food safety specialist about cases of shellfish illness when they responded to a relevant call. However, this step could easily be forgotten if such calls were received by DPIC during a period of heavy volume or in the midst of managing more critical cases. Indeed, ASIRCS was developed after an informal audit of the old system found that half of relevant cases had not been flagged for review by BCCDC food safety specialists.

Once a computer is correctly programmed to do a task, it is incapable of making human errors. Using computers to reliably detect and report aberrations in large data is fundamental to modern public health surveillance. Even so, our analyses demonstrate that automated systems are not infallible, and that ongoing evaluation is required to ensure optimal performance. Furthermore, ongoing training of poison specialists is required to ensure that the quality of poison control data meets the needs of public health surveillance. Specifically, poison specialists must understand how any given surveillance system uses the data they collect so they have it in mind when recording details about a relevant case. This requires clear and ongoing communication between the poison specialists, developers, and end users to optimize and improve the accuracy and performance of operational systems. Retrospective application of the ASIRCS algorithm to complete data identified 11 cases that failed to generate alerts, of which 6 would have been referred to regional health authorities. These findings highlight the need for code developed using the principles of computer science rather those of data analysis, such that errors are caught and reported as they occur. The code for ASIRCS now performs a self-check every hour confirming its status, and automatically sends an email to technicians.

Furthermore, the addition of “seafood” to the keyword list identified 11 new cases, 1 of which would have been referred to the responsible health authority. This keyword has been permanently added to the list, and work is ongoing to improve the free text search. For example, ASIRCS does not currently account for common misspellings of the keywords because the BCCDC does not have adequate in-house experience or capacity in this area of data science [55].

As public concern over poison exposures grows, developing and upgrading methods for real-time automated alerting on poison center data can improve responses to public health threats. The model we describe for surveillance systems developed in R provides a flexible and adaptable framework with the potential to identify new threats as they emerge. Examples include the following: newly introduced household products, such as laundry pods [56]; drugs, such as paramethoxymethamphetamine [30]; commercially available toxicants, such as pesticides [57,58]; and illnesses resulting from intentional or unintentional exposures at a single site or across a large area, such as oil spills [59]. At present, the BCCDC is using a similar system to detect potential overdoses from exposure to fentanyl, which is part of a larger provincial effort to address the ongoing public health emergency in BC [60]. The BCCDC is also supporting a work-in-progress collaboration between all Canadian poison centers to establish a national database similar to that of the NPDS, which would vastly improve national surveillance of poisonings.

**Figure 3:**
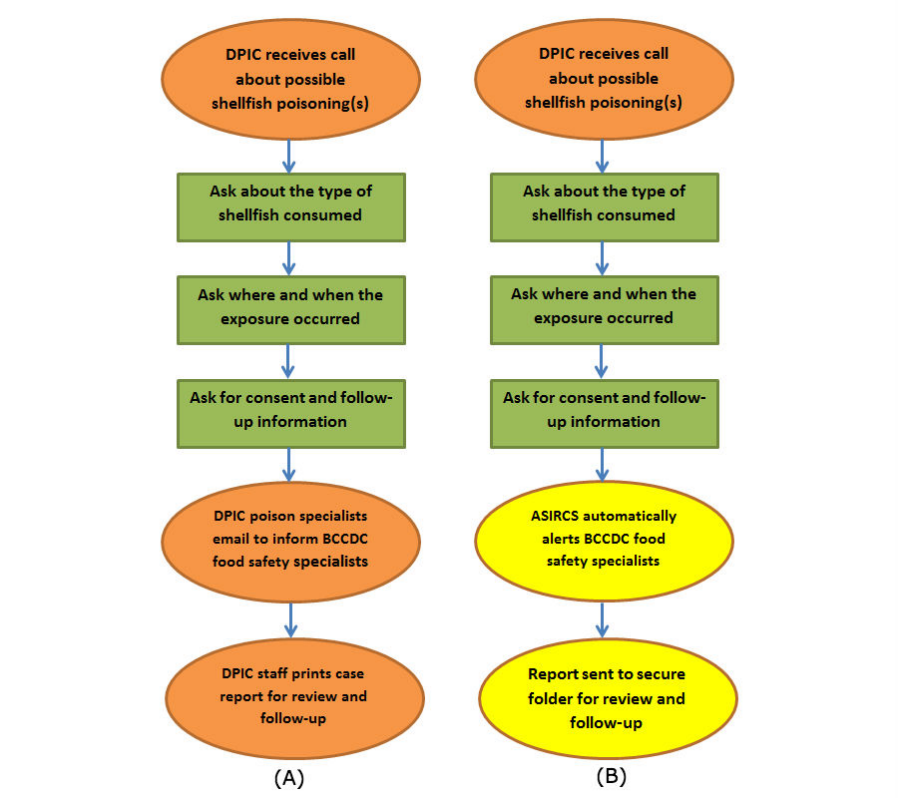
An illustration of the old (A) and new (B) procedures alerting British Columbia Centre for Disease Control (BCCDC) food safety specialists about calls made to the Drug and Poison Information Centre (DPIC) related to shellfish consumption. The new procedure (B) uses the automatic alerting system for illness related to consumption of shellfish (ASIRCS) running in the R statistical computing environment.

The surveillance utility and value of DPIC data are evident here. During this study, there were two large shellfish outbreaks in BC because of (1) *VP* in May to September 2015 and (2) norovirus in November 2016, both of which were evident in ASIRCS alerts (Figures 2 and 3). However, the use of poison control data is limited by a number of factors. First, DPIC case data can be incomplete depending on the nature of the call. As seen with ASIRCS, there were 6 cases where public health action could not be taken because of the absence of follow-up contact information. Second, exposures are often self-reported and may not represent true shellfish poisoning incidents; so, the system relies on the precision of DPIC poison specialists when gathering information about each case. This is especially challenging because BC shellfish can be contaminated with viruses, bacteria, and toxins that may have overlapping symptoms. Fortunately, other surveillance mechanisms allow us to contextualize information from DPIC using other environmental and laboratory data [61]. Unfortunately, complementary data from the national shellfish biotoxin monitoring program are not readily available for public health surveillance at this time. Finally, surveillance systems such as ASIRCS require considerable technical expertise to develop and maintain, and some poison centers may not have the necessary human resources.

The core function of DPIC and other poison centers is to serve and educate the public and health care providers. Although this foundation remains unchanged, there is increasing pressure on poison centers to integrate their data with public health surveillance mandates. Here, we have described the development and application of a novel system of data collected by DPIC poison specialists made readily and quickly accessible to the BCCDC food safety specialist for improved public health. By combining rich poison data with the analytic power of the R computing environment, the possibilities for future work are almost limitless.

## Appendix

Multimedia Appendix 1 A sample of the report (based on falsified data) generated by the alerting system for illnesses related to consumption of shellfish for review by food safety specialists. BC: British Columbia; DPIC: Drug and Poison Information Centre.

## Abbreviations

ASIRCS: alerting system for illness related to consumption of shellfish
ASP: amnesic shellfish poisoning
BC: British Columbia
BCCDC: British Columbia Centre for Disease Control
DPIC: Drug and Poison Information Centre
DSP: diarrhetic shellfish poisoning
NPDS: National Poison Data System
PSP: paralytic shellfish poisoning
VP: Vibrio parahaemolyticus
VDLE: Visual Dotlab Enterprise
