# Supplementary figures and images for: Near-Real-Time Surveillance of Illnesses Related to Shellfish Consumption in British Columbia: Analysis of Poison Center Data
Source: JMIR Public Health Surveill. 2018 Feb 23;4(1):e17. doi: 10.2196/publichealth.8944 (PMC5845107; doi:10.2196/publichealth.8944)

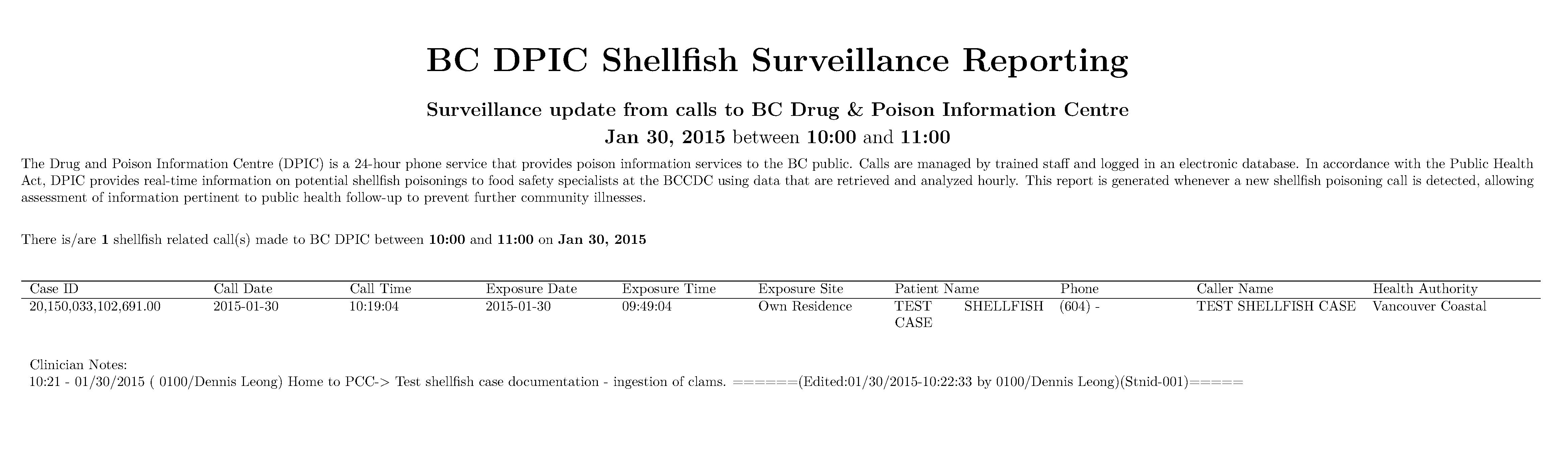

Supplement: Multimedia Appendix 1 [file publichealth_v4i1e17_app1.png]
